# Joint optimization of headway and number of stops for bilateral bus rapid transit

**DOI:** 10.1371/journal.pone.0300286

**Published:** 2024-03-13

**Authors:** Rongrong Guo, Francisco Antunes, Jin Zhang, Jingcai Yu, Wenquan Li

**Affiliations:** 1 School of Transportation, Southeast University, Nanjing, China; 2 Centre for Informatics and Systems of the University of Coimbra, University of Coimbra, Coimbra, Portugal; University of Valencia: Universitat de Valencia, SPAIN

## Abstract

The bilateral Bus Rapid Transit (BRT) system is a kind of BRT system in which the stops are located in the middle of the transit lane. By simultaneously serving transit lines in opposite directions, it is particularly designed to save space resources and enhance service quality. To improve the operational efficiency of the bilateral BRT, this paper optimizes the operational performance of bilateral BRT with elastic demand. The objective is to minimize the generalized time cost per passenger of the system by jointly optimizing the headway and number of stops of bilateral BRT. The cost includes the agency operating and user travel. The optimal design model is formulated as a mixed-integer program and solved using a fuzzy analytic hierarchy process (FAHP) and a genetic algorithm (GA). We conduct a case study and sensitivity analysis to show the effectiveness and reliability of the proposed approach. We conclude that the optimized minimum generalized cost per passenger is lower than the actual case for all demand levels, especially at off-peak hours, by about 22.5%. In addition, we find that the weights of agency and user costs have the most significant impact on headway, whereas the influence of walking, vehicle speed, and route length is minimal. In contrast, the optimal number of BRT stops is mostly influenced by the route length, and walking speed has essentially no effect on the optimal number of stops. Finally, we find that the generalized cost per passenger at peak hours is 10% to 15% smaller than at off-peak hours in various scenarios.

## 1. Introduction

Public transportation (PT) is one of the crucial infrastructures of urban and social development. The development of public transport is a necessary measure to lead the sustainable development of urban transport in the future. Bus rapid transit (BRT) is an emerging mode of urban PT that can achieve large capacity, speed, and service quality at relatively low cost by combining segregated bus lanes that are typically median aligned with off-board fare collection, level boarding, bus priority at intersections, and other quality-of-service elements [1]. They apply rail-like infrastructure and operations to bus systems with offerings that can include high service quality, special bus lanes, intelligent systems, etc. Therefore, it can meet the transportation requirements of large capacity, high speed, and high service quality at a relatively low cost [2, 3].

BRT systems currently operate in about 200 countries in Latin America, Asia, Europe, North America, Africa, and Oceania [4]. Operation ways are a BRT system’s central elements and its infrastructural foundation [5], which primarily includes mixed traffic lanes, curb bus lanes, median bus lanes, and bus-only streets. Each type of operation way has some advantages and disadvantages, and different regions use different ways of operation. In this case, the operation of the median bus lanes can also be referred to as a mid-street bilateral BRT system, where one stop can serve BRT vehicles operating on both sides simultaneously. Obviously, one of the primary benefits of such a system is reduced construction costs, mostly due to the utilization of existing medians [6]. This form of busway is widely used, for example, in South America, Japan, and China. Many operation experiences, such as TransMilenio [7], BRT Line 1 in Beijing [8], etc., show that the bilateral BRT system has made interesting progress in terms of travel time savings, increased operational speed and punctuality, high passenger satisfaction, and high capacity.

The planning of a BRT system affects the surrounding environment it serves. In the system, the mental health and ability of the driver can have an impact on the performance and efficiency of the system, e.g., factors such as job insecurity and high stress affect driver behavior and reduce the operational efficiency of the system [9], whereas reducing the amount of incoming information and avoiding fatigued driving can increase their efficiency and improve the system performance [10, 11]. In addition to the driver, the design of the system also has an impact on the operational performance of the system, which is the focus of this research. There are several factors that go into the design of a system, such as network structure, service area, headway, number of vehicle stops, and cost-effectiveness. Forming a relatively complete BRT operation network on urban roads can improve the operation efficiency and service quality of public transport in a short time [12]. The forms of network structure can be linear corridors, grids, radial, etc. The linear corridor structure that has been used in practice, such as the BRT1 line in Jinan.

Regardless of the form of network structure, the headway of transit systems is a factor of utmost importance, affecting the reliability of BRT service and having a great impact on the efficiency of vehicle operation and the attractiveness of passenger travel [13, 14]. Typically, headway directly affects the waiting time of passengers, which is often reported as the largest costly component of total passenger costs [15, 16]. Therefore, many studies focus on passenger waiting time when optimizing headway or departure interval/frequency. For example, Newell [17] and Zhao et al. [18] only optimize the departure interval with the goal of minimizing the waiting time of passengers. In contrast, although Han and Wilson [19] developed a similar work, the authors considered the route selection behavior of passengers. Based on Newell’s [17] scheduling policy, Wirasinghe [20] used an analytical method to optimize headway that minimizes not only passenger waiting time but also scheduling costs. Also considering passenger and vehicle scheduling, Shang et al. [21] develop an extended vehicle scheduling model to obtain the minimum waiting time for both passenger and vehicle departure intervals. In addition to considering the waiting time, Leiva et al. [22] present an optimization approach that minimizes some costs in terms of in-vehicle travel time and operator cost. They establish a variety of optimization models to determine the optimal transit departure frequency under different models. On the other hand, Larrain et al. [23] optimize vehicle scheduling under the condition of congested and non-congested lines and use a heuristic algorithm to solve the optimal departure frequency of transit lines. Feng et al. [24] establish a multi-objective optimization model of transit departure interval with the largest transit operation profit, the smallest passenger transfer waiting time, and the smallest passenger travel cost. Most of these studies above focus on the traditional transit service, whereas only a few study bilateral BRT system services. Moreover, passenger perspective metrics, such as waiting time, are oftentimes considered within the optimization models but rarely analyze the optimization from the perspective of the whole system cost.

When optimizing the operation of transit, although some studies optimized only headway, more studies focus on the joint optimization of headway with some other factors, including load factor, vehicle type, stop, fare, etc. [25–29]. Typically, many studies consider elastic demand when designing fares because passengers will be sensitive to fares [30–33]. Although several studies optimize both fares and headways, in some zones, transit fares are set at a very low flat rate when the transit is more public-spirited, which does not change with demand or vehicle operations, such as Jinan, Nanjing, and Dalian in China. In this case, passengers are less sensitive to fares and more concerned with headways. However, few studies take into account elastic demand when only designing the operation of vehicles. In those situations, the actual demand of passengers at the same potential demand level is not constant across scenarios. Therefore, we should consider the elastic demand function in the operational design.

In addition, the number of vehicle stops on BRT lines is designed as one of the decision variables because the number affects the efficiency of vehicle operation and service quality, especially at different time periods or demand densities (peak and off-peak). At first, the agency may choose to strategically deploy more stops during peak hours when demand density is highest. Conversely, during off-peak hours when demand density is lower, the agency might opt to close certain stops to allocate resources efficiently. Thus, the utilization of specific stops in the BRT system is contingent upon fluctuating demand levels and different time periods. In this case, the bilateral BRT system has a clear advantage over the regular BRTs since the same stop in the former can serve both sides of the operating vehicles.

In this context, this paper takes the bilateral BRT system as the research object and constructs a joint optimization model of headway and the number of vehicle stops with elastic demand. The objective of the optimization model is to obtain the minimum generalized time cost per passenger, including agency operating costs and user time costs. The research aims to help decision-makers better formulate the operation of bilateral BRT, improve the service level and operational efficiency of urban BRT, especially during peak and off-peak hours, and enhance the attractiveness of urban public transport. The main contributions of this study include:

1. Develop a mixed-integer nonlinear optimization model to obtain the optimal headway and number of stops of bilateral BRT to minimize generalized cost per passenger.
2. Propose a linear elastic demand function to derive the actual demand densities based on potential demand densities, which in turn leads to a more realistic generalized time cost per passenger.
3. Design the fuzzy analytic hierarchy process (FAHP) to assign the weight of agency cost and user cost in the generalized cost per passenger.
4. Conduct experiments and compare cases to demonstrate the validity and properties of the optimization model.

The rest of the paper is organized as follows. Section 2 formally describes the bilateral BRT system. Then, Section 3 devises the optimization model for the problem. The results of the case study and numerical experiments are presented and discussed in Section 4. Finally, Section 5 summarizes the main findings of this paper and makes recommendations for future research.

## 2. System description

**Fig 1:**
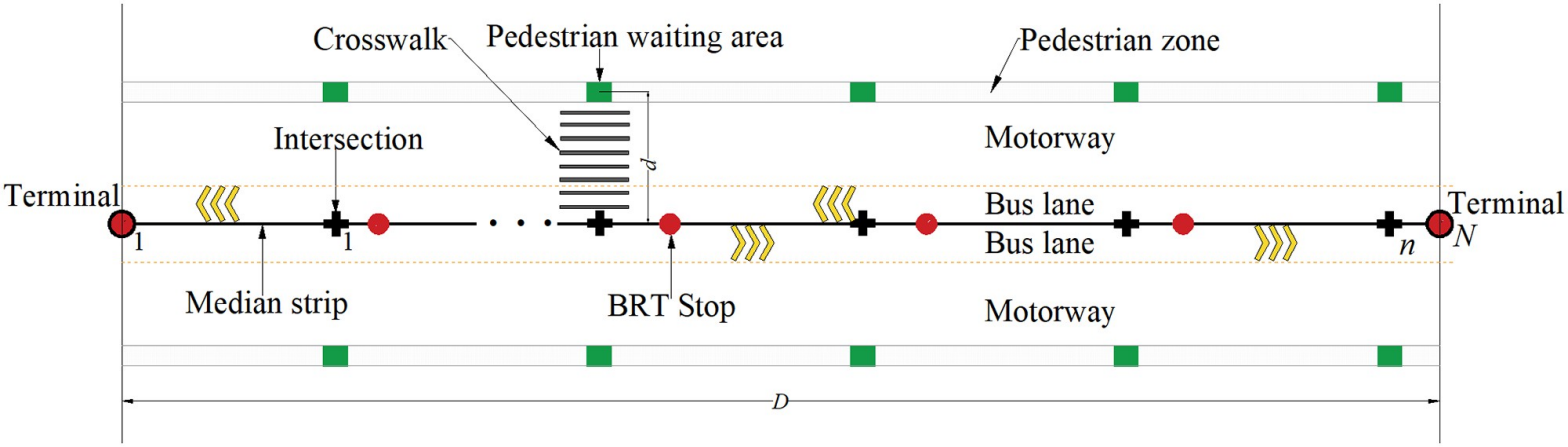
Overview of the bilateral Bus Rapid Transit system.

In this section, we provide the essential details for the system under study. The route of the bilateral BRT system is located in the middle of the motorway. As shown in Fig 1, we use rectilinear movement to approximate a realistic road network (e.g., BRT-1 line in Jinan, China) and consider the length *D* (km) of the service area, delimited by two terminals. There are BRT-dedicated bus lanes on both sides of the median strip, and the stops of the bilateral BRT system are located on the median strip of the motorway. One BRT stop can serve vehicles and passengers on both sides of the median strip simultaneously. For simplicity, we set equidistant stops and equidistant intersections on the route. *N* and *n* denote the number of BRT stops and intersections, respectively. The crosswalk distance from the intersection to the pedestrian area is expressed as *d* (km). We define the passenger walking distance as the length of passengers walking away from the origin of the pedestrian zone to the nearest BRT stop by passing through the pedestrian zone, the crosswalk, and the distance between the stop and intersection in sequence. The headway for the BRT lines is denoted as *H* (h), with *H* ∈ (0,1] being one of the design variables. The vehicle cruising speed and passenger walking speed are denoted as *v*_*b*_ (km/h) and *v*_*p*_ (km/h), respectively.

To simplify the bilateral BRT system and analysis, the following four assumptions are introduced:

- Assumption 1 –The operation of private cars is prohibited in the bus lane, and the BRT vehicles operate without taking into account the effects of unexpected events.
- Assumption 2 –Vehicle capacity can meet passenger demand. Passengers who fail to take the previous bus will take the next BRT vehicle in the line.
- Assumption 3 –The acceleration and deceleration of BRT vehicles are, by default, the same for each start and stop. Ignoring the propagation time of the starting wave of BRT vehicles at the intersection.
- Assumption 4 –The pedestrian zone generates *λ*_0_ potential passenger trips per hour per kilometer. Passengers’ origins are uniformly and independently distributed on the sidewalk.

## 3. Modeling

The purpose of the design problem is to determine the optimal values for the decision variables, *H* and *N*, such that the generalized cost per passenger of the bilateral BRT system is minimized. The generalized cost per passenger includes agency operation costs and user costs. For the reader’s convenience, the key notations used in the paper are listed as follows:

*A* = Average total walking time per passenger (h);
*C* = Average signal cycle of intersection (min);
*D* = Length of the BRT service (km);
*d* = Average crosswalk half width from intersection to pedestrian area (m);
*f* = Fare of BRT (Chinese Yuan, CNY);
*H* = Headway of the bilateral BRT system (h);
*L* = Expected total distance traveled per hour of vehicles (km/h);
*l*_*o*_ = Distance from passenger getting off to leaving the stop (m);
*M* = Expected total fleet size per hour of vehicles (veh);
*N* = Number of vehicle stops of BRT lines;
*n* = Number of intersections;
*Q* = Average arrival number of vehicles (veh/h);
*s* = The distance between two adjacent streets (km)
*S* = Average saturated flow rate of vehicles leaving intersection (veh/h);
*T* = Average total in-vehicle travel time per passenger (h);
*t*_*bw*_ = Average vehicle waiting time at intersections (h);
*t*_*pw*_ = Average passenger waiting time at intersections (h);
*U* = Utility function;
*v*_*b*_ = Vehicles’ cruising speed (km/h);
*v*_*p*_ = Walking speed (km/h);
*W* = Average total waiting time per passenger (h);
*Z* = Generalized time cost per passenger (h);
*α* = Average green ratio in BRT operation direction;
*β* = Average green ratio of passengers passing through the crosswalk;
*δ*_*L*_ = Operating cost per vehicle distance (CNY/veh·km);
*δ*_*M*_ = Operating cost per vehicle hour (CNY/veh·h);
*θ* = The parameter reflects the passenger’s perception of the travel cost;
*λ* = Actual number of passengers per hour per kilometer (pax/h/km);
*λ*_0_ = Potential number of passengers per hour per kilometer (pax/h/km);
*μ* = Value of time (CNY/h);
*τ*_lost_ = Time lost per stop or intersection due to deceleration and acceleration (s);
*τ*_stop_ = Dwelling time (s);
*ψ* = The parameter of the elastic demand function;
*ω*_1_,*ω*_2_ = Weight of agency operating costs or user costs.

### 3.1. Agency costs

The total agency operation costs are determined by the expected total vehicle distance traveled per hour of operation, *L*, and the expected total fleet size in operation, *M* [34].

The distance traveled by a BRT vehicle in one round trip is 2*D* within the research area, and the headway of the vehicle is *H*. Thus, the expected vehicle distance traveled in one hour is *L* = 2*D*⁄*H*.

The time required for BRT vehicles to cover the distance in one hour includes the expected travel time *L*⁄*v*_*b*_, expected waiting time at intersections *t*_*bw*_ and BRT stops *τ*_stop_, and the time lost per stop due to deceleration and acceleration *τ*_lost_. According to Assumption 1, BRT vehicles generally operate in the dedicated lane in a non-saturated state. Let *Q* denote the average arrival number of vehicles and *S* the average saturation flow rate of vehicles leaving the intersection. Based on steady-state theory [35], vehicle waiting time at intersections contains three components: the first component is the normal phase waiting, the second component is the random delay, and the third component is derived from traffic simulation tests. Thus, the expected waiting time at the intersection is given by

$$t_{bw}=\frac{C{\left(1-\alpha \right)}^{2}}{2\left(1-Q/S\right)}+\frac{Q^{2}/{\left(\alpha S\right)}^{2}}{2Q\left[1-Q/\left(\alpha S\right)\right]}-0.65\sqrt[{3}]{\frac{C}{Q^{2}}}{\left(\frac{Q}{\alpha S}\right)}^{2+5\alpha },$$ (1)

where *C* is the average signal cycle of intersection and *α* is the green ratio in BRT vehicle operation direction. Thus, the expected fleet size in one hour can be calculated as

$$M=\frac{L}{v_{b}}+\frac{2\left(n-1\right)t_{bw}+2N\tau_{\text{stop}}+4\left(N+n\right)\tau_{\text{lost}}}{H}.$$ (2)

### 3.2. User costs

The user costs are associated with the total time for passengers to travel from their origins to their destinations. As seen in works such as Qiu et al. [36], the user costs function includes three main components: walking time *A*, waiting time *W*, and in-vehicle travel time *T*.

**Fig 2:**
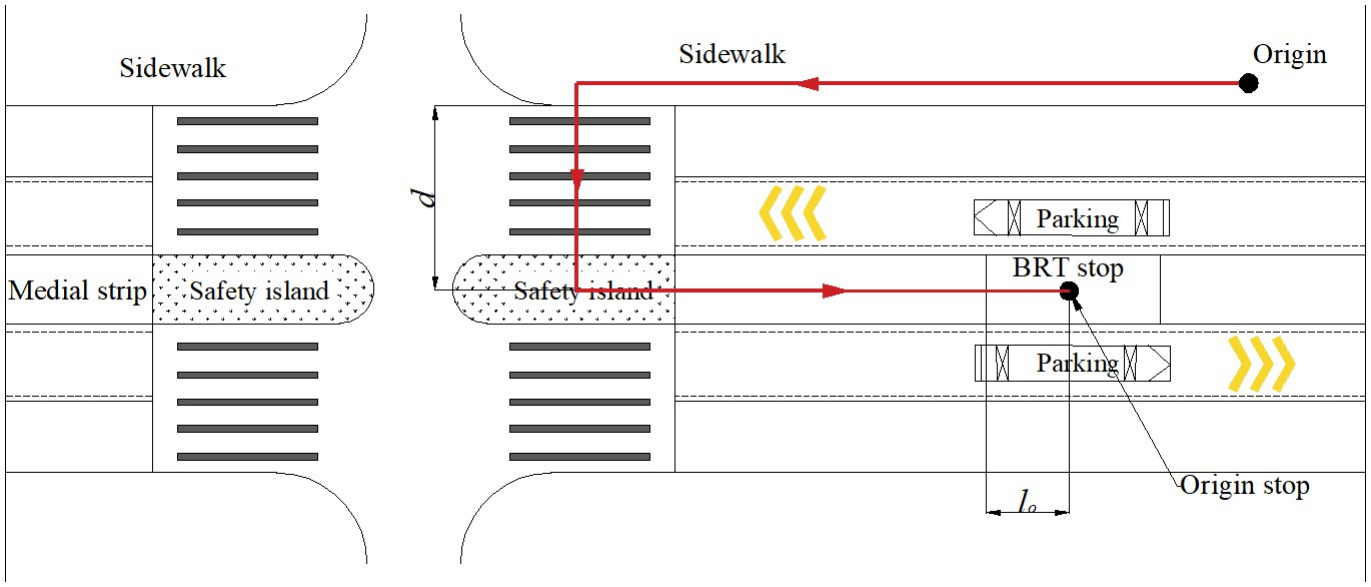
Illustration of the walking route for passengers arriving at the stop.

The walking time is the time from the origin to the nearest BRT stop (origin stop), as shown in Fig 2. However, it is not always possible for passengers to cross the pedestrian crossing immediately when they walk to the intersection. In other words, not all passengers arriving at the intersection within the hour will encounter a green light. Therefore, the average waiting time of passengers at the intersection should be included in the walking time *t*_*pw*_. The direction of the passenger crossing the roadway is perpendicular to the direction of vehicle operation. Let *β* denote the green ratio for passengers crossing the road. The number of arriving passengers who can immediately cross the intersection can be approximated by *λβ*, so that their waiting time at the intersection is zero. The number of passengers who cannot immediately cross is *λ* (1 − *β*), and the average waiting time is about half of the red light time due to the uniformity of vehicle arrival at the stop, i.e., *C* (1 − *β*)⁄2. Therefore, the average passenger waiting time at one intersection is expressed as

$$t_{pw}=\frac{\lambda \left(1-\beta \right)\times \frac{C\left(1-\beta \right)}{2}+\lambda \beta \times 0}{\lambda }=\frac{C{\left(1-\beta \right)}^{2}}{2}.$$ (3)

Let *d*_*i*_ represents half of the length of the pedestrian crossing at the *i*-th intersection and the average, *l*_0_ denotes the distance of passengers passing the platform, and *l*_*d*_ denotes the remaining distance of the walking process, which can be derived according to Assumption 4. Thus, the expected walking time per passenger is

$$A=\frac{l_{0}+l_{d}}{v_{p}}+\frac{\sum_{i=1}^{N}d_{i}}{nv_{p}}+t_{\text{pw}}=\frac{l_{0}+d}{v_{p}}+\frac{1}{2}\left(\left|\frac{D}{N-1}-\frac{D}{n-1}\right|+\frac{D}{N-1}\right)/v_{p}+\frac{C{\left(1-\beta \right)}^{2}}{2}.$$ (4)

The expected waiting time per passenger at each stop is approximately half of the headway time [37, 38]. Therefore, the waiting time for passengers at the origin stop is given by *W* = *H*⁄2.

Then, the average in-vehicle travel time of passengers is calculated using the method of Aldaihani et al. [37]. The range of an operating BRT route is divided equally into (*N*-1) zones. Let *D*_*N*−1_ denote the cumulative distance of all possible outcomes of passenger trips on the BRT in (*n*-1) zones. Thus, the average in-vehicle travel distance per passenger can be calculated as

$$d_{N-1}=\frac{D_{N-1}D}{\sum_{i=1}^{N-2}2i}=\frac{\frac{D}{N-1}\sum_{i=1}^{N-2}2i\left(N-1-i\right)}{\sum_{i=1}^{N-2}2i}.$$ (5)

Therefore, the average in-vehicle travel distance per passenger is given by

$$T=\frac{d_{N-1}}{v_{b}}=\frac{D\sum_{i=1}^{N-2}2i\left(N-1-i\right)}{v_{\text{b}}\left(N-1\right)\sum_{i=1}^{N-2}2i}.$$ (6)

### 3.3. Elastic demand

The demand density of BRT services is affected by in-vehicle time, waiting time, and fare [38]. In addition to these time cost components, walking time also affects whether a user chooses BRT service. Therefore, we believe that the demand density of BRT service is also affected by walking time. Overall, the optimization of transit operation depends on the demand of passengers, and the demand of passengers is elastic, which is subject to the utility of the user cost, including walking time, waiting time, in-vehicle time, and fare. Accordingly, following Sun and Szeto [27], we adopt a linear elastic demand function

$$\lambda =\lambda_{0}-\psi \left(-\frac{1}{\theta }\text{ln}\left(\text{exp}\left(-\theta \cdot U\right)\right)\right),$$ (7)

where *U* is the utility function, *U* = (*A* + *W* + *T*)*μ* + *f*, *λ*_0_ is the potential demand. Both *ψ* and *θ* are the parameters of the elastic function reflecting the passenger perception of travel cost. The default value for *ψ* and *θ* are 0.5 and 0.1, respectively [27].

### 3.4. Formulation

We now present the optimal design model. To define the objective function consistently, the distance traveled by the BRT vehicles and the fleet size should be converted into travel time equivalents. We used the method of Daganzo to calculate the conversion factor [39]. Let *δ*_*L*_, *δ*_*M*_ and *μ* be the agency operation cost per vehicle distance, the agency cost per vehicle hour, and the average monetary value of one passenger hour, respectively. Then, the conversion factor of the corresponding operating costs into equivalent travel time per passenger can be expressed as *T*_*L*_ = *δ*_*L*_⁄(*λDμ*) and *T*_*M*_ = *δ*_*M*_⁄(*λDμ*). Thus, the minimum generalized cost per passenger optimization model designed is represented as

$$\text{min}\;Z\left(H,N\right)=\omega_{1}\left(T_{L}L+T_{M}M\right)+\omega_{2}\left(W+A+T\right).$$ (8)

$$\text{s.t.}\;0<H\leq 1,N\in \left\{1,2,\ldots ,\left\lfloor \frac{D}{s}\right\rfloor \right\}$$ (9)

where *ω*_1_ and *ω*_2_ are the weights of agency operating costs or user costs, respectively. Rather than assigning them directly, we calculate the weighting values for agency costs and user costs in a reasonable way. Constraint (9) is the definitional constraint for the two decision variables, where *N* is an integer variable. Although *N* has no natural upper bound, for simplicity, we set its upper bound to ⌊*D*⁄*s*⌋, which is quite loose [40].

The fuzzy analytic hierarchy process (FAHP) methodology is usually used to assign weights [41]. From the perspective of time cost, the more passengers get off at a stop, the longer the waiting time for passengers still on the bus, indicating that the vehicle has been running for a longer time. Therefore, it is reasonable to consider the ratio *ρ* of the number of passengers who do not get off the vehicle to the number of those who get off the vehicle when arriving at a stop as a comparison of the importance of passengers and vehicles *a*_1_ and *a*_2_ (see Table 1) [42]. If *a*_*i*_ is compared with *a*_*j*_ to give *a*_*ij*_, and *a*_*j*_ is compared with *a*_*i*_ to give *a*_*ji*_ = 1 − *a*_*ij*_, so a fuzzy reciprocal comparison matrix can be constructed, such that

$$a_{\text{matrix}}=\left[\begin{array}{l} a_{11},a_{12}\\ a_{21},a_{22} \end{array}\right]$$ (10)

**Table 1:** Fuzzy scale value.

***ρ***	[0,0.3]	(0.3,0.5]	(0.5,0.7]	(0.7,0.9]	(0.9,2]	(2,4]	(4,6]	(6,8]	(8,+∞)
***a*_*ij*_**	0.1	0.2	0.3	0.4	0.5	0.6	0.7	0.8	0.9

Then, based on the single hierarchical arrangement, the weighting factor can be calculated,

$$\omega_{i}=\frac{1}{k}-\frac{1}{2\eta }+\frac{1}{k\eta }\sum_{1}^{k}a_{ik},\begin{array}{cc} & i=\left\{1,2\right\} \end{array},$$ (11)

where *k* is the order of the fuzzy consistency matrix, *k* = 2, and *η* is the unit of importance difference measured between agency operation and user costs, *η* ≥ (*k* − 1)⁄2. A small *η* means that the decision-maker requires a large difference in importance between agency operation costs and user costs. We set *η* to the default value of 0.7.

## 4. Case study

In this section, we first introduce a heuristic-based algorithm method to solve the optimization model. Following this, case and sensitivity experiments are performed to analyze the properties of the optimization model.

### 4.1. Solution method

The optimal design problem of the bilateral BRT system is a mixed-integer optimization problem. The usual method to solve such problems is via heuristic algorithms [34]. We choose a global optimization method called the elitist genetic algorithm (EGA). We use the Python library ‘geneticalgorithm’ for implementing the EGA. The steps of the solution algorithm are shown as follows.

Step 1. Encode the headway (*H*) and the number of stops (*N*) of BRT as the chromosome and set the range of chromosomes.

Step 2. Generate initial populations randomly within the variable range and the initial generalized cost per passenger through fitness function, Eq (8).

Step 3. Set the parameters of the genetic algorithm (refer to the Python library ‘geneticalgorithm’), including the population size, the number of variables, crossover probability, mutation probability, and the maximum number of iterations.

Step 4. Implement selection operation using a proportional selection operator. The smaller the fitness function, the closer to the optimal solution and the higher the probability that the selection can enter the next generation.

Step 5. Determine the number of elites in the population. The default value is 0.01 (i.e., 1 percent).

Step 6. Given the crossover probability, determine the chance that a pre-existing solution will pass its genome (aka trait) to a new solution.

Step 7. Set mutation probabilities for mutation operations to generate new chromosomes to maintain population diversity.

Step 8. Decode each individual to the real value, calculate the optimal local solution and the fitness value of each individual, and then save the result.

Step 9. Determine if termination conditions are satisfied, and set the maximum number of iterations to 1000. If it is satisfied, the calculation results are outputted. If not, return to Step 4.

The EGA parameters are set as follows:

algorithm_param={'max_num_iteration':1000,'population_size':100,'mutation_probability':0.1,'elit_ratio':0.01,'crossover_probability':0.5,'parents_portion':0.5,'crossover_type':'uniform','max_iteration_without_improv':None}

### 4.2. Cost comparison

**Fig 3:**
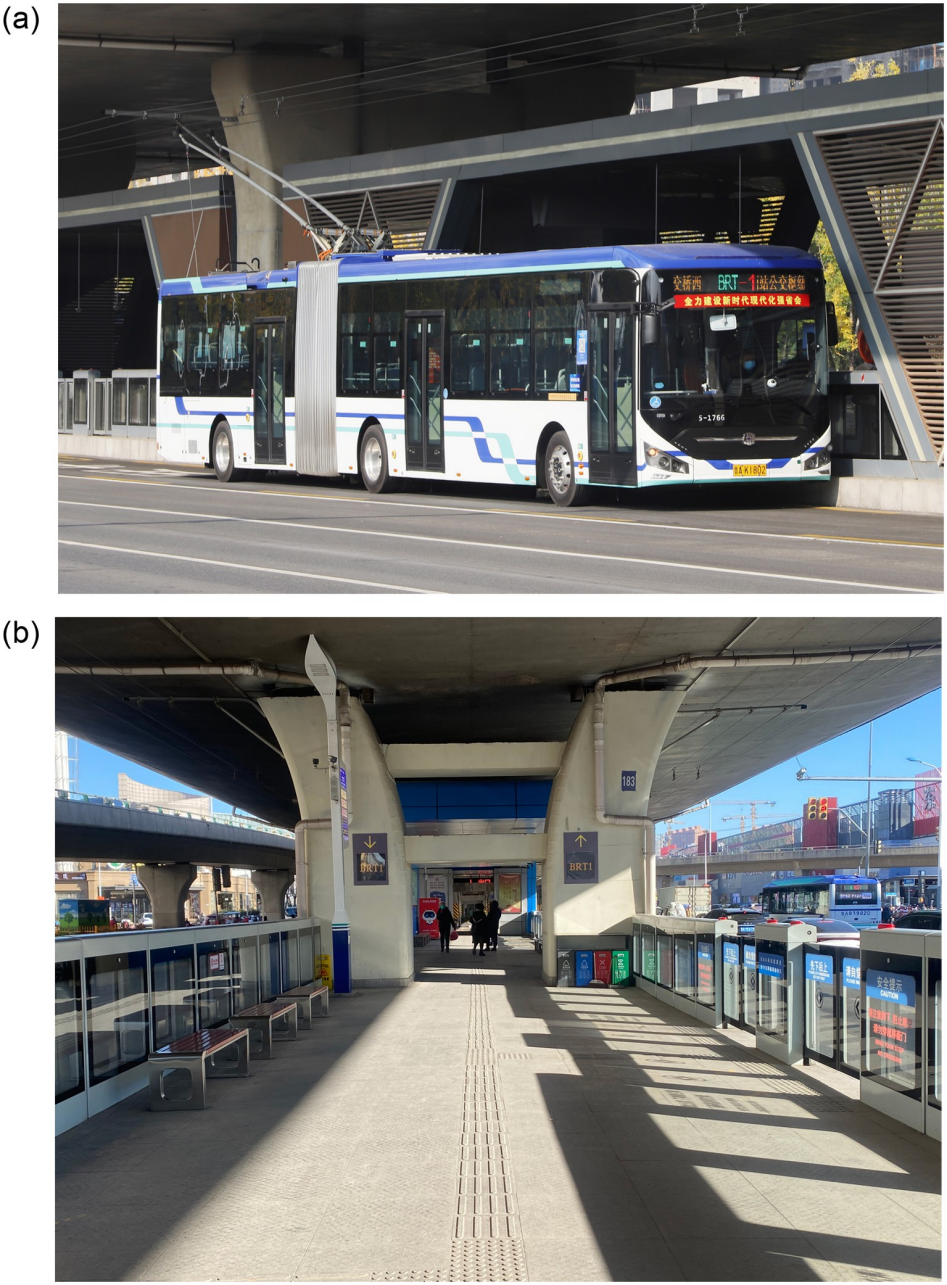
Display of the bilateral BRT line in Jinan.

The BRT-1 line on Beiyuan Street in Jinan (a city in China) is a bilateral transit line in the middle of the road (see Fig 3). It is equipped with dedicated bus lanes on both sides of the median strip. Therefore, one BRT stop can serve BRT lines on both sides at the same time. The BRT-1 route (11 km in total, *s* = 0.2 km) runs in a straight line and is similar to the operating scenario of the BRT system studied in this paper. We first calculate the default weights *ω*_1_ and *ω*_2_. After investigation, the ratio of the average number of passengers who do not get off to the number of passengers who get off the vehicle when arriving at a stop *ρ* = 5, thus *a*_12_ = 0.7 and *a*_21_ = 1 − *a*_12_ = 0.3 (see Table 1). *a*_11_ is the importance of *a*_1_ compared to *a*_1_, which is clearly equally important, i.e., *ρ* = 1 and *a*_11_ = 0.5. Similarly, *a*_22_ = 0.5. Thus, the fuzzy judgment matrix is $\left[\begin{array}{l} 0.5,0.7\\ 0.3,0.5 \end{array}\right]$. Finally, based on Eq (11), the weight values for operator and user are *ω*_1_ = 1⁄2 − 1⁄1.4 + 1⁄1.4 × (0.5 + 0.7) = 0.64 and *ω*_2_ = 1⁄2 − 1⁄1.4 + 1⁄1.4 × (0.5 + 0.3) = 0.36, respectively. Based on the average income level of residents in the city, the value of time is set to 20 CNY/h. The other default values of the input parameters are listed in Table 2.

**Table 2:** Values of input parameters.

**Notation**	*D*	*s*_*o*_	*α*	*β*	*v*_*b*_	*C*	*S*	*δ*_*M*_
**Value**	10 km	25 m	0.32	0.31	25 km/h	3min	0.4veh/s	20 CNY/veh·h
**Notation**	*d*	*s*	*τ*_lost_	*τ*_stop_	*v*_*p*_	*μ*	*Q*	*δ*_*L*_
**Value**	20 m	50 m	2 s	10 s	5 km/h	20 CNY/h	0.05veh/s	2 CNY/veh·km

**Fig 4:**
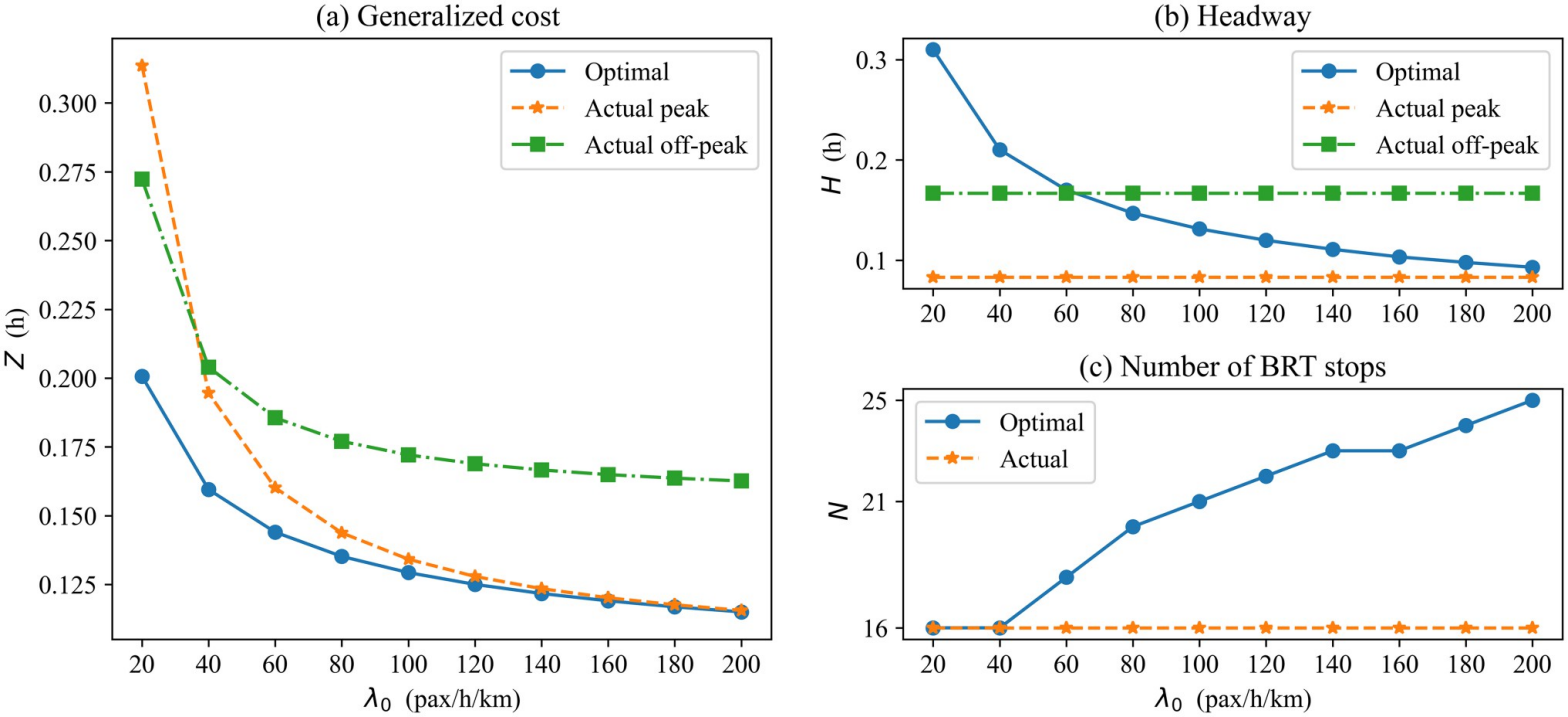
Comparison of results under variable potential demand.

Fig 4 illustrates the actual situation of BRT-1 lines at off-peak and peak hours compared to the optimized results under different potential demand levels. The trends of the minimum generalized time cost per passenger are presented in Fig 4a. As expected, all three costs decrease with increasing demand. We can see that the optimal generalized cost per passenger is smaller than the actual cost under all potential demand levels. This also illustrates the validity of the optimization model. Fig 4b shows the optimal headway of BRT-1, which can be smaller than the actual headway when demand is low during off-peak hours (e.g., *λ*_0_ = 40 pax/h/km), and the optimal number of stops is the same as the actual number of stops (Fig 4c). During peak hours, the optimal headway of BRT is consistently larger than the actual headway (Fig 4b), and the optimal number of BRT stops is more than the actual number of stops (Fig 4c). In other words, this shows that adjacent BRT vehicles can operate at larger headway, and the BRT vehicles should run shorter distances between adjacent stops, i.e., fewer buses and more stops. This comparison test illustrates that the actual number of stops is reasonable during off-peak hours but less during peak hours. In practice, only smaller headway is considered for BRT-1 to meet peak hour passenger demand, but adjusting headway alone does not improve walking time per passenger. The reason is that the walking time is the time from the sidewalk to the nearest BRT stop (see Fig 2) and does not vary with the headway of BRT. If the number of stops is jointly optimized, the walking time can be improved, which in turn reduces the generalized time cost per passenger.

Table 3 lists the actual and optimal results for the BRT-1. The actual survey found that the potential demand *λ*_0_ is about 60 pax/h/km during the off-peak hours, while the demand during the peak hours is about 160 pax/h/km (estimated potential demand per hour per kilometer based on the distance between two adjacent stops and the number of boardings at each stop). For a more efficient analysis of actual and optimized scenarios, the corresponding potential demand values are used for comparison during the off-peak hours and the peak hours, respectively. Compared with the actual situation, the optimized generalized cost per passenger remains essentially unchanged at peak hours and reduced by about 22% at off-peak hours. Correspondingly, the optimal headway increases by about 19% at peak hours and remains essentially unchanged at off-peak hours, and the number of stops increases by about 43% at peak hours and 12% at off-peak hours. This suggests that operators can increase the number of stops and headway appropriately during different periods.

**Table 3:** The results from actual conditions and optimal model results.

Demand *λ*_0_ (pax/h/km)	Optimal			Actual peak hours			Actual off-peak hours		
	*H*(h)	*N*	*Z* (h)	*H*(h)	*N*	*Z* (h)	*H*(h)	*N*	*Z* (h)
60	0.170	18	0.144	—			0.167	16	0.186
160	0.103	23	0.119	0.083	16	0.120	—		

### 4.3. Sensitivity analysis

**Fig 5:**
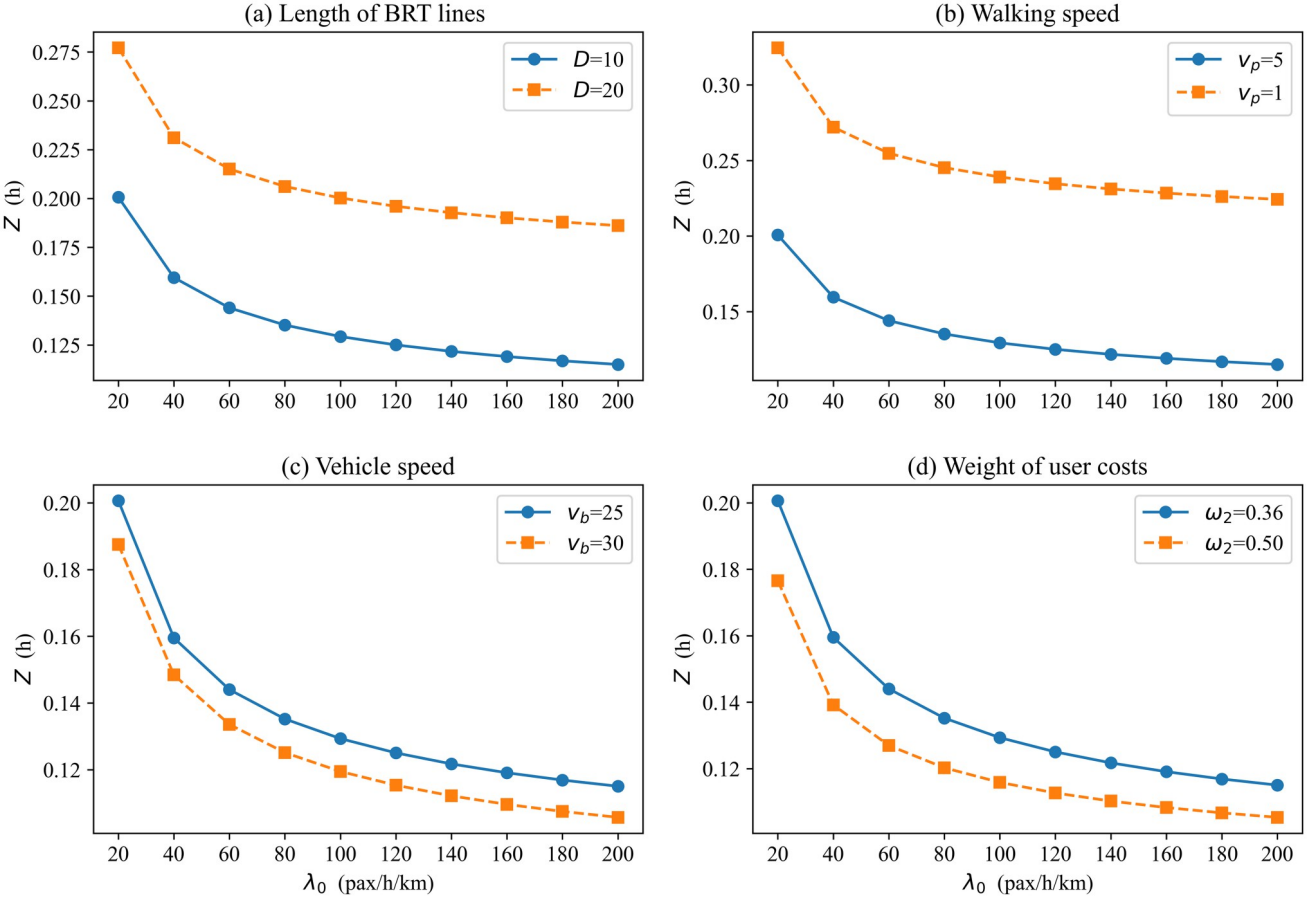
Minimum generalized cost vs. demand with different input parameters.

**Fig 6:**
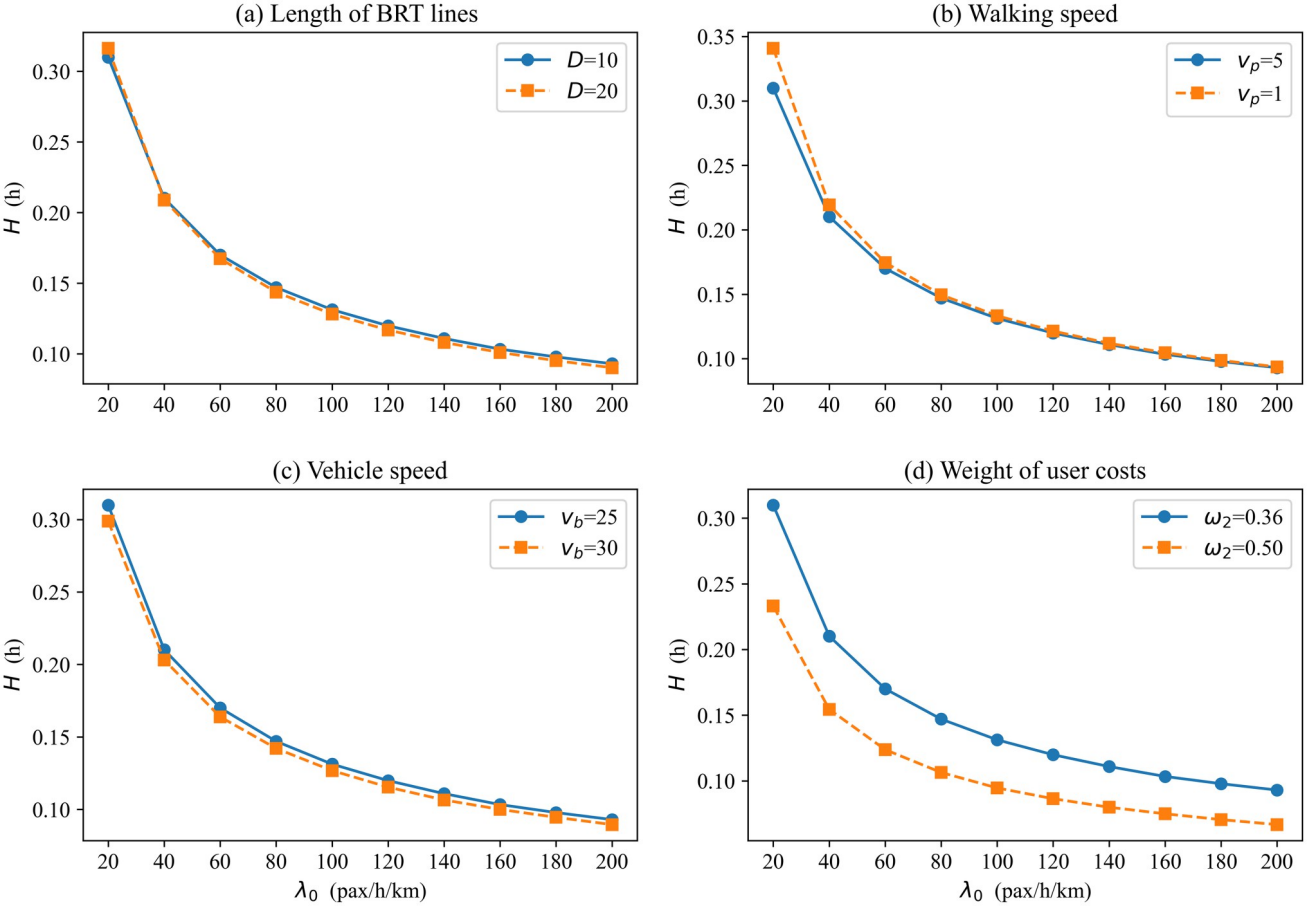
Optimal headway vs. demand with different input parameters.

**Fig 7:**
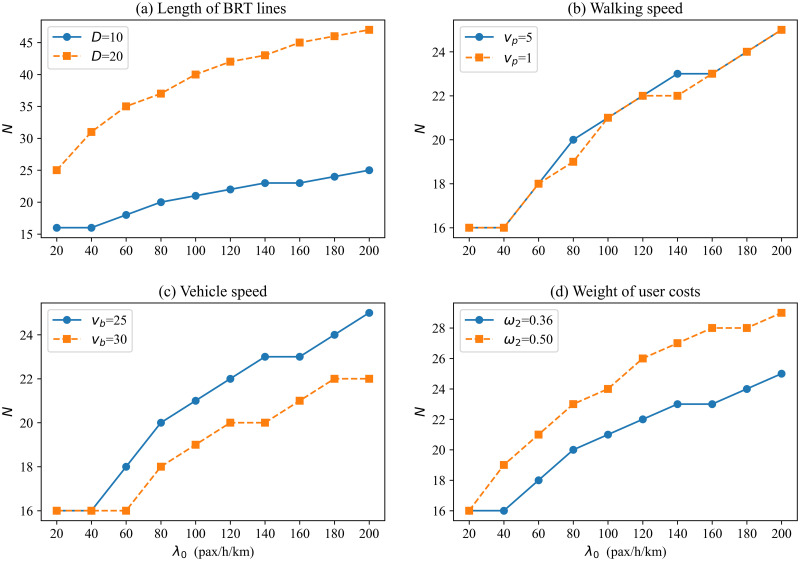
Optimal number of stops vs. demand with different input parameters.

In this section, we conduct a sensitivity analysis to examine how the optimal result of BRT-1 systems is affected by key input parameters. The variation of the minimum generalized cost per passenger is shown in Fig 5 when changing the key input parameters, including BRT line length, walking speed, vehicle speed, and weights of user costs. Figs 6 and 7 present the headway and number of BRT stops variation for different scenarios.

Fig 5a plots the minimum generalized cost per passenger versus demand level for two different lengths of transit routes: *D* = 10 km (a short route); *D* = 20 km (a mid-route) [39]. When the potential demand is 20 pax/h/km, the generalized time cost per passenger is about 38% higher on the mid-route (0.277 h) than on the short route (0.201 h). When potential demand is 200 pax/h/km, the generalized cost per passenger is about 62% higher on the mid-route (0.186 h) than on the short route (0.115 h). Therefore, the generalized time cost per passenger under the short route trends down faster as demand increases, and managers may prefer shorter routes. The reason is the higher the demand, the longer the vehicle operating delays and the longer the average passenger travel time under mid-route conditions. Therefore, it is not advisable to operate excessively long routes when potential densities are high. Although the generalized cost per passenger of mid-route is higher than that in the short route, managers may also choose to operate a bilateral BRT system under mid-route conditions if the range of generalized cost increases is acceptable. Obviously, the number of stops also increases with the increase in route length (Fig 7a). By comparison, it is found that the gap between adjacent station spacing decreases with increasing demand at different lengths. This indicates that station spacing should be longer at a low demand level on a medium-length route (e.g., increases by 28% when *λ*_0_ = 20), while the same station spacing can be set for high demand levels. For example, when the demand is 20, regardless of short or medium route length, Fig 6a shows that the curves of the optimal headway are similar in both cases. That is, the length of the BRT line affects the number of stops but has little effect on the headway of the BRT vehicles. This is the combined effect of headway and number of stops for vehicles in the bilateral BRT system.

Fig 5b and 5c compare the generalized cost per passenger curves for different walking speeds and vehicle speeds. Low walking(vehicle) speed may be used in undesirable situations, such as extreme weather [37, 43]. As expected, both lower walking and vehicle speeds result in higher costs. Figs 6b and 7b display that the low walking speed has little effect on the number of stops and headway. Similarly, Fig 6c shows that low vehicle speed has little effect on the headway, but the effect on the number of stops is more pronounced. The higher the vehicle speed, the lower the optimal number of stops. In addition, the poorer environment also affects the travel patterns of passengers. The test results show that low speed leads to a reduction in actual demand. Therefore, a good service environment can also increase actual demand.

Fig 5d shows the effect of user cost weights on the generalized cost per passenger. We find that the weight of the user is inversely proportional to the generalized cost per passenger, and the generalized cost per passenger decreases as the weight increases. This is because as the weight of users increases, the agency has to consider the user travel cost more. Accordingly, the headway is reduced (Fig 6d), and the number of stops is increased (Fig 7d) to derive a smaller user cost.

**Fig 8:**
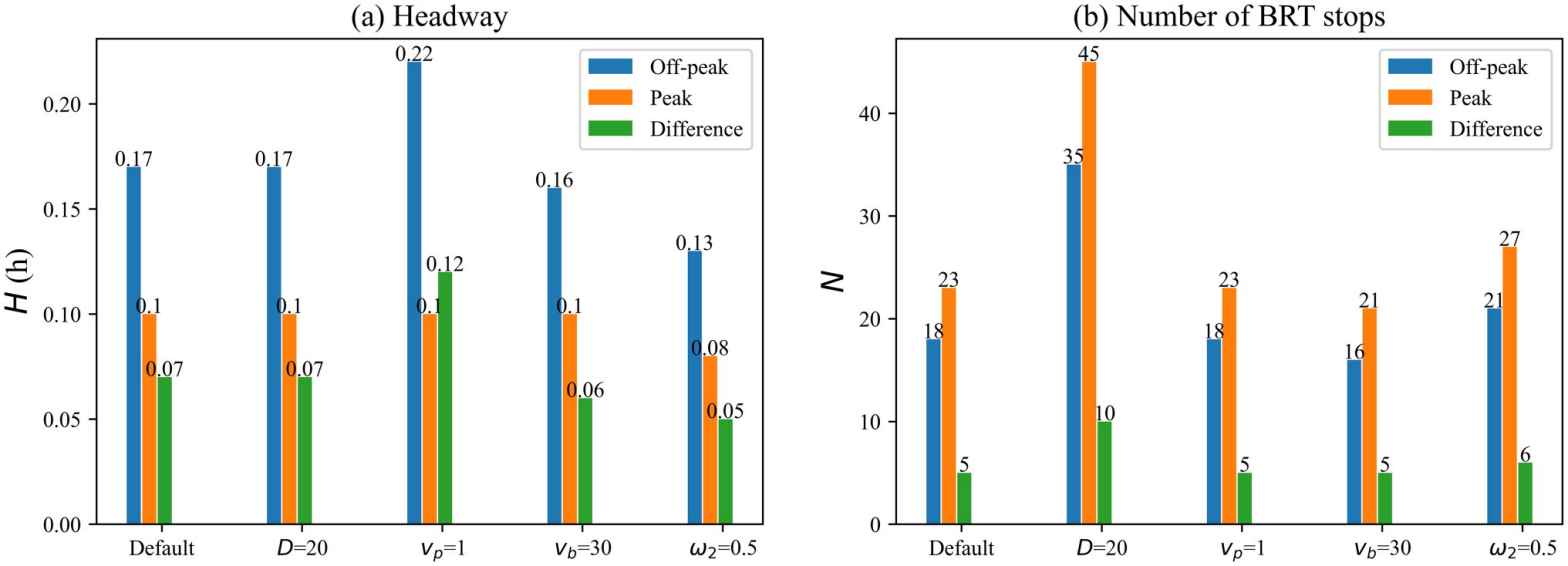
Comparison of headway and number of stops under off-peak and peak hours.

Finally, we analyze the change in headway and the number of stops during the off-peak and peak hours. Similarly, we set when the potential demand is 60, it is set as an off-peak hour, and when the demand is 160, it is a peak hour. As shown in Fig 8a, the optimal headway during peak hours is approximately 40%-50% less than during off-peak hours. The optimal number of BRT stops increases by about 25%-30% (Fig 8b). This indicates that the frequency of BRT vehicle departures in an hour increases during peak hours, while station spacing is reduced to ensure the efficiency of BRT vehicle operations. Correspondingly, the minimum generalized cost per passenger during peak hours is reduced by about 10%-15% compared to off-peak hours. Comparing the effect of different parameters on the variation of the optimal result, we can see that the walking speed (walking environment) has a more significant effect on the difference in headway at peak and off-peak hours, while the weight value of cost has the least effect. For the differences in the number of BRT stops, the effect of route length is the most significant.

### 4.4. Discussion

In comparison with existing studies that consider the generalized cost per passenger as the objective of optimizing transportation systems [34, 38–40], the trend of the curve of the cost with demand in these studies is the same: all decreases as demand increases, which is also consistent with the results of this paper. However, the focus of attention is not the same in these studies, e.g., some studies focus on the design of the service area, some focus on the site selection or route design, and others only focus on the optimization of headway or number of stops alone, but rarely consider the synergy of the two. The reason for this is that in practice, the number of stops in an optimized BRT line has been determined and is usually not changed, so transit agencies react to changes in demand density during different time periods (peak and off-peak) only by adjusting the headway. However, headway adjustments alone do not improve the cost of walking for passengers. In addition, bilateral BRT systems with stops located in the middle of the roadway can serve BRT vehicles operating on both sides at the same time, which is more advantageous in practice. Therefore, this study attempts to collaboratively optimize the headway and stops of the bilateral BRT system for the purpose of operational optimization design. As expected, the proposed model has a smaller generalized cost compared to the actual.

To further analyze the properties of the proposed model, following the related studies [39], this study chose to analyze the different line lengths, walking speeds, and vehicle speeds for BRT services. In addition, we considered the impact of agency and user weight assignments on the results. The reason for considering this weighting parameter is that agencies in different regions have different focuses when operating bilateral BRT systems. For example, in China, where the BRT system is controlled by the government, the users are the focus of attention compared to the agency’s operating costs. On the other hand, BRT systems in some countries are controlled by private companies (e.g., the United States and Denmark), and the costs and benefits of operation are emphasized along with the users. Therefore, a reasonable weight allocation is necessary. This research designs a rational allocation of weights to balance the agency and users, which is one of the main contributions of this study. Moreover, management agencies can also set the weights according to the specific objectives.

## 5. Conclusion

Bus Rapid Transit (BRT) is seen as a viable solution to traffic congestion and resource conservation because it is faster than regular bus service and less expensive to build than rail. Therefore, researchers are constantly optimizing the BRT operation system to improve the quality of transit service. This paper aims to reduce the generalized time cost of the bilateral BRT system by jointly optimizing the headway and number of stops of bilateral bus rapid transit. With the objective of minimum generalized cost, a mixed-integer nonlinear program model is constructed. The cost includes the agency’s operating costs and the user costs. The model is considered from both agency and user perspectives, and weights are assigned to both. A fuzzy analytic hierarchy process is used to reasonably assign the weight values of agency costs and user costs, and a heuristic algorithm is used to solve the model. We selected the BRT-1 line in Jinan, China, as a practical case for simulation tests and compared the actual and optimized results during off-peak and peak hours. Finally, we perform a sensitivity analysis on the model to evaluate the reliability of the optimization model. The main findings from these tests are summarized below. The main findings from these experiments are summarised as follows:

1. As demand increases, the optimal headway of BRT decreases while the optimal number of stops increases. Compared to the actual operation, the optimized minimum cost is smaller for different demands and time periods, especially at off-peak hours, and the generalized cost is reduced by approximately 22.5%. Correspondingly, the optimal headway and number of stops are increased both in the off-peak and peak hours.
2. Weight assignment has the most significant effect on the optimal headway, while the length of the BRT route, walking, and vehicle speed have minor effects on the optimal spacing.
3. A good walking and vehicle operating environment (high walking speed) can reduce generalized costs and increase the amount of actual demand. However, the walking environment has essentially no effect on the number of BRT stops.
4. Compared to the optimal number of BRT stops, the optimal headway is more influenced by peak and off-peak hours in various input parameter scenarios. And the generalized cost during peak hours is about 15% smaller than during off-peak hours.

The results also show that the optimal number of stops is larger during peak hours than during off-peak hours, approximately 28%, which can provide the agency with decision help in implementing the construction. The agency can deploy stops based on the demand density during peak hours while choosing to close some intermediate stops based on the demand density during off-peak hours.

There are several limitations to the research in this paper that can be studied in more depth in the future. First, the bilateral BRT system studied in this paper used several simplifying assumptions to optimize generalized cost for linear structures. To support more types of BRT systems in practice, future research could relax these assumptions to consider more realistic scenarios and more structural routes, such as radial structures or hybrid structures. Next, this research did not consider roadside/unilateral BRT systems. Intuitively, when transit stops are located on the curb, passengers walk less distance, and vehicles run more distance, which affects the associated cost components. Therefore, another possible direction for future research is to change the object of research and analyze roadside/unilateral BRT systems, then compare them with the bilateral BRT system. Last but not least, this study focuses on the operational optimization of the BRT system and does not consider facility costs and driver behavior during the construction process. However, facility costs and driver behavior can also affect the operational performance of the system to some extent. Therefore, optimization of related construction costs and driver behavior in the system can be considered in future studies.

## Supporting information

S1 File (DOCX)
