# Supplementary material for: Joint optimization of headway and number of stops for bilateral bus rapid transit
Source: PLoS One. 2024 Mar 13;19(3):e0300286. doi: 10.1371/journal.pone.0300286 (PMC10936851; doi:10.1371/journal.pone.0300286)
Supplement: S1 File — (DOCX) [file pone.0300286.s001.docx]

Supporting information

**The source code for reproducing the experiments in this study are available in the 1st author’s GitHub repository:** [ronguo2/BRT-Test (github.com)](https://github.com/ronguo2/BRT-Test)**.**
